# Mesh Fixation to the Sacral Promontory From the Left Side of the Mesosigmoid: Two Cases of Robot‐Assisted Sacrocolpopexy for Persistent Descending Mesocolon

**DOI:** 10.1002/iju5.70039

**Published:** 2025-04-29

**Authors:** Kojiro Tanabe, Yasuno Takahashi, Yuki Takahashi, Ryohei Hashimoto, Yoshiko Oyama, Yuko Hatakeyama, Hitoshi Niikura

**Affiliations:** ^1^ Department of Obstetrics and Gynecology Sendai Medical Center Sendai Miyagi Japan; ^2^ Department of Obstetrics and Gynecology Graduate School of Medicine, Tohoku University Sendai Miyagi Japan; ^3^ Department of Obstetrics and Gynecology Kyoritsu Narashinodai Hospital Chiba Japan

**Keywords:** mesh fixation to the sacral promontory from the left side of the mesosigmoid, pelvic organ prolapse, persistent descending mesocolon, robot‐assisted sacrocolpopexy

## Abstract

**Introduction:**

Persistent descending mesocolon is a congenital fixation abnormality where the left‐sided colon deviates medially. When significantly displaced to the right, it may affect sacrocolpopexy. We report two cases of persistent descending mesocolon in which robot‐assisted sacrocolpopexy was successfully performed with mesh fixation to the sacral promontory from the left side of the mesosigmoid.

**Case Presentation:**

Two patients with pelvic organ prolapse underwent robot‐assisted sacrocolpopexy. In both cases, the sigmoid colon was displaced to the right, making exposure of the sacral promontory from the right side of the mesosigmoid challenging. Therefore, the sacral promontory was exposed and mesh fixed from the left side of the mesosigmoid. The postoperative course was uneventful.

**Conclusion:**

When the sigmoid colon is displaced rightward due to persistent descending mesocolon, sacral promontory fixation from the left mesosigmoid may be a safe, feasible option.

**Abbreviations**
CT: computed tomography
PDM: persistent descending mesocolon
POP: pelvic organ prolapse
POP‐Q: pelvic organ prolapse quantification
RASC: robot‐assisted sacrocolpopexy

**Summary**

- Mesh fixation from the left side of the mesosigmoid may be a safe and feasible option when the sigmoid is displaced to the right due to persistent descending mesocolon (PDM).
- Before sacrocolpopexy, PDM should be considered, and the position of the intestine should be carefully assessed through imaging.

## 1 Introduction

Persistent descending mesocolon (PDM) is a congenital fixation abnormality where the left‐sided colon deviates medially due to incomplete fixation to the retroperitoneum [1, 2]. Most cases of PDM are asymptomatic [2], with an incidental detection rate of 1.2%–2.4% during gastrointestinal surgery [3, 4]. The extent of medial deformation and adhesion to surrounding structures varies widely, and no standardized definition exists for PDM. In cases where the left‐sided colon is significantly displaced to the right, it can affect sacrocolpopexy, which exposes the sacral promontory from the right side of the mesosigmoid. Here, we present two cases of robot‐assisted sacrocolpopexy (RASC) in which the mesh was successfully fixed to the sacral promontory from the left side of the mesosigmoid due to PDM.

## 2 Case Presentation

### 2.1 Case 1

**FIGURE 1:**
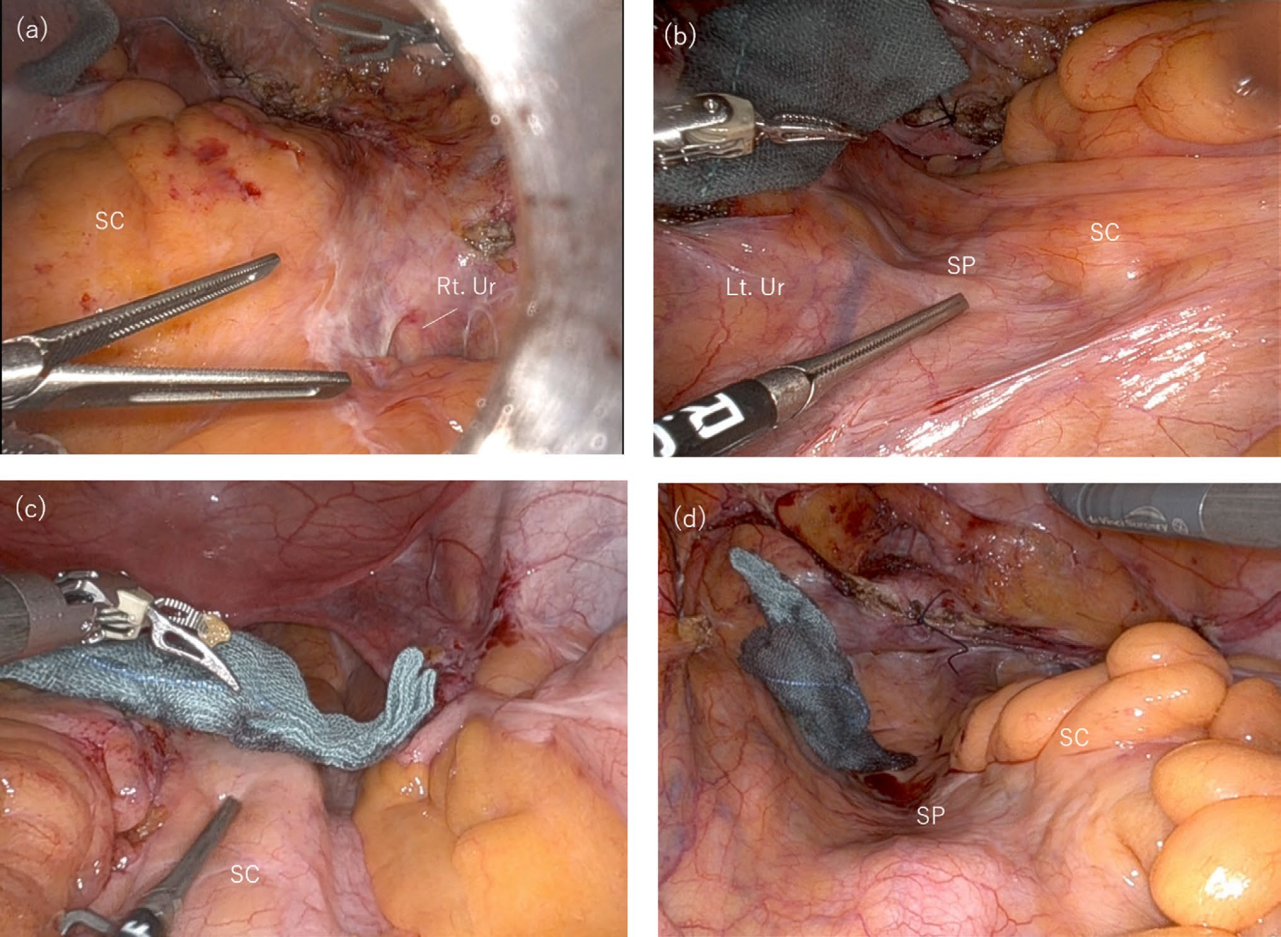
Intraoperative findings. (a) Case 1: The sigmoid colon is adherent to the right pelvic wall, with the right side of the sacral promontory covered by the mesosigmoid. (b) Case 1: The sacral promontory is visible through the peritoneum to the left of the mesosigmoid. (c) Case 2: The sigmoid colon is displaced to the right, narrowing the space for exposure of the sacral promontory and complicating dissection. (d) Case 2: The sacral promontory is visible through the peritoneum to the left of the mesosigmoid. Lt, left; Rt, right; SC, sigmoid colon; SP, sacral promontory; Ur, ureter.

**FIGURE 2:**
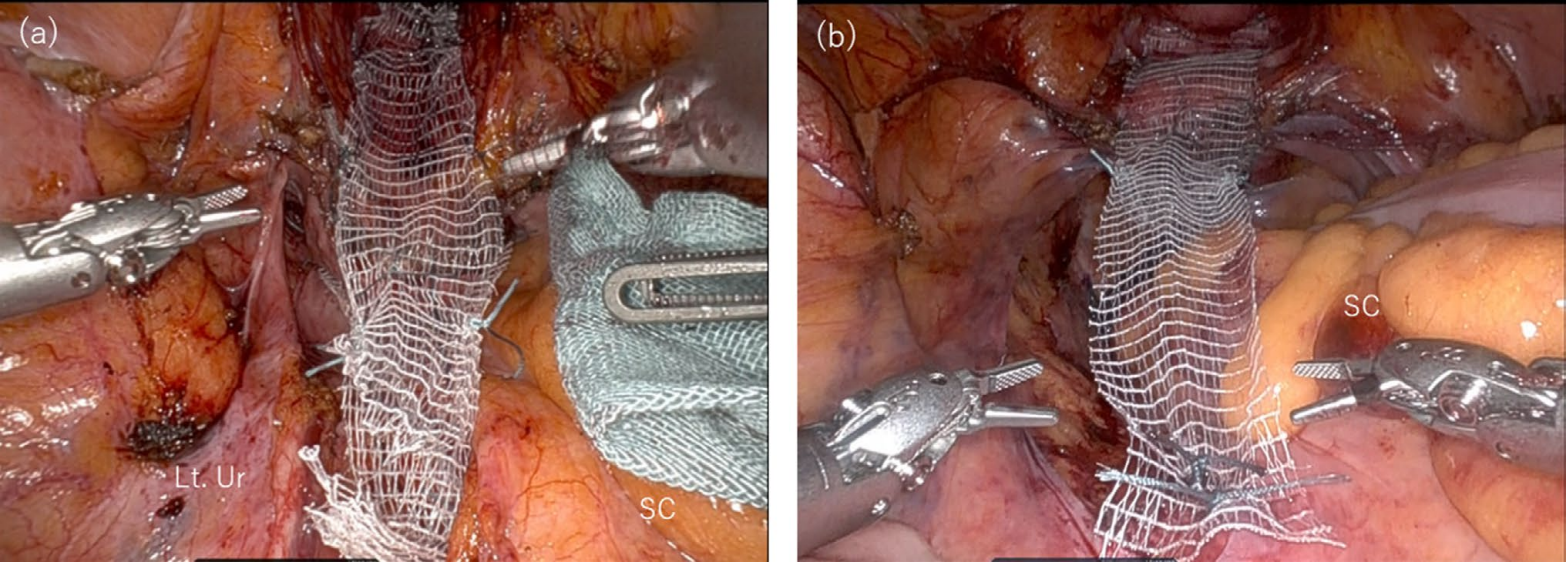
Mesh fixation to sacral promontory from the left side of mesosigmoid. (a) Case 1, (b) Case 2: After fixation of the mesh to the sacral promontory. Lt, left; SC, sigmoid colon; Ur, ureter.

A 77‐year‐old woman (gravida 3, no prior abdominal surgeries) was diagnosed with pelvic organ prolapse quantification (POP‐Q) stage III (Aa +2, Ba +2, C + 3, Ap +2, Bp +2). The patient is an active agricultural worker and is expected to continue experiencing abdominal pain post‐operatively. Considering the lower recurrence rate of RASC compared to native tissue repair, she opted for and underwent RASC. Intraoperatively, adhesions between the sigmoid mesocolon, right pelvic wall, and ileocolic area were observed, leading to a diagnosis of PDM. The right sacral promontory was obscured by adhesions (Figure 1a), whereas the left sacral promontory was visible through the peritoneum (Figure 1b). Due to the complexity of adhesiolysis, an excision was made in the peritoneum on the left side of the mesosigmoid to expose the sacral promontory. Rightward retraction of the sigmoid colon provided sufficient space and facilitated exposure. An anchoring suture was placed on the anterior longitudinal ligament, and the peritoneal incision was extended to the Douglas pouch. A mesh was used to reinforce the anterior and posterior vaginal walls and was subsequently fixed to the sacral promontory (Figure 2a). The mesh arms were retroperitonealized with continuous suturing. The total operative time was 3 h 51 min, with minimal blood loss. The patient was discharged on postoperative Day 4. Nine months postoperatively, she remained symptom‐free with no recurrence of POP.

### 2.2 Case 2

A 72‐year‐old woman (gravida 2, with no history of abdominal surgery) was diagnosed with POP‐Q stage III (Aa +2, Ba +2, C 0, Ap −3, and Bp −1). The patient requested RASC due to being sexually active and the procedure's lower recurrence rate. Intraoperatively, the sigmoid colon was displaced to the right, restricting exposure of the sacral promontory from the right side of the mesosigmoid (Figure 1c). However, a wide area of the sacral promontory was visible through the peritoneum on the left side (Figure 1d), allowing exposure and mesh fixation to be performed from the left side, following the same procedure as in Case 1 (Figure 2b). The operative time was 3 h 43 min, with minimal blood loss. The patient was discharged on postoperative Day 4 and remained symptom‐free with no recurrence of POP at the 6‐month follow‐up.

**FIGURE 3:**
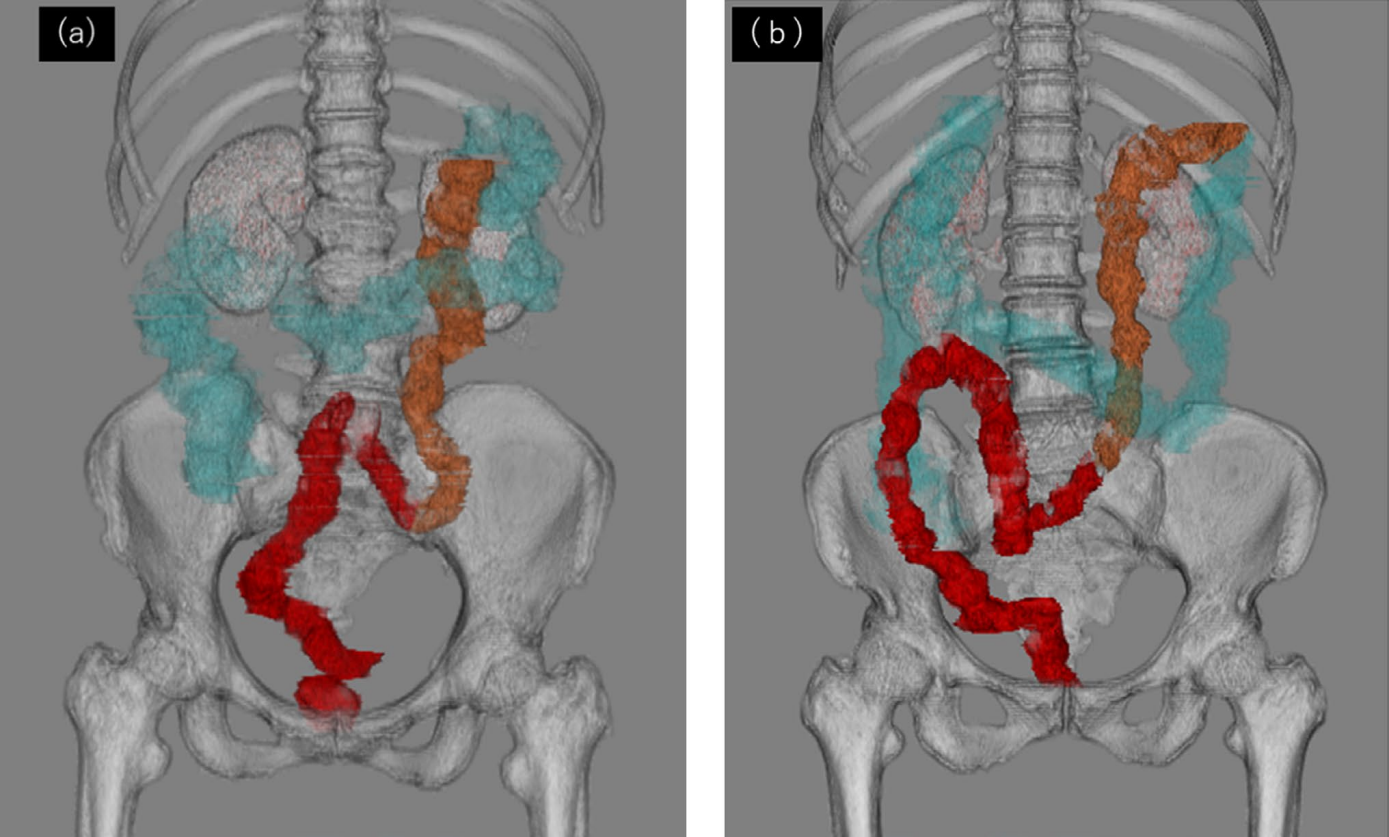
Three‐dimensional computed tomography (3D‐CT) volume rendering image of the colon. (a) Case 1, (b) Case 2: The descending and sigmoid colon are displaced rightward due to persistent descending mesocolon (PDM), making right‐sided exposure of the sacral promontory difficult. Blue: ascending and transverse colon; Yellow: descending colon; Red: sigmoid colon and rectum.

**FIGURE 4:**
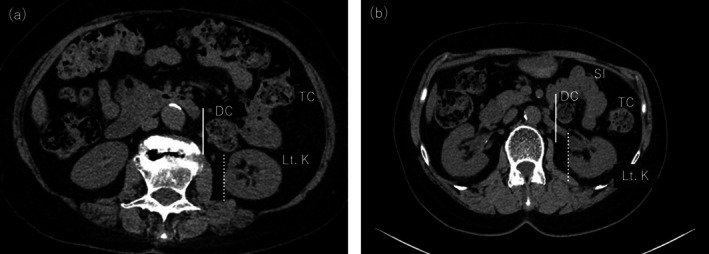
Axial CT images at the level of the left kidney. (a) Case 1, (b) Case 2: The descending colon is medially displaced. The right edge of the descending colon (solid line) is located more medially than the right edge of the left kidney (dotted line). DC, descending colon; Lt.K, left kidney; SI, small intestine; TC, transverse colon.

In both cases, 3D‐CT volume rendering images of the colon were created based on the CT images obtained preoperatively, and rightward displacement of the descending and sigmoid colon was confirmed (Figure 3) Additionally, the right edge of the descending colon was medial to the right edge of the left kidney (Figure 4). These findings were consistent with those of PDM.

## 3 Discussion

PDM is a congenital fixation abnormality in which the left‐sided colon deviates medially due to incomplete fixation to the retroperitoneum [1, 2]. In PDM, anomalies in the branching of the inferior mesenteric artery and mesenteric shortening may be observed [5]. While PDM is recognized in gastrointestinal surgery [3, 4, 5], reports on its impact during sacrocolpopexy are rare. To the best of our knowledge, only one report in the literature written in English describes a case of laparoscopic sacrocolpopexy for a patient with PDM [6]. We reported two cases of POP with PDM, successfully treated with RASC using mesh fixation to the sacral promontory from the left side of the mesosigmoid. In both the previous and present cases, rightward displacement of the sigmoid colon impeded the sacral promontory from the right side of the mesosigmoid. Consequently, the sacral promontory was exposed, and mesh fixation was performed from the left side of the mesosigmoid.

Exposure of the sacral promontory from the left side of the mesosigmoid requires caution due to its unfamiliarity. Additionally, the left common iliac vein runs dorsomedially to the left common iliac artery, posing a risk near the promontory. However, by retracting the sigmoid colon to the right during sacral promontory exposure, the surrounding area can be adequately visualized, allowing for safer dissection. While caution is warranted, the risks associated with a right‐sided approach may outweigh those of a left‐sided approach in cases of PDM. The limited dissection space on the right side often necessitates mobilization of the sigmoid colon, which may be complicated by vascular anomalies or mesenteric shortening [5]. For non‐gastrointestinal surgeons, such dissection can be technically demanding and potentially dangerous.

Mesh fixation from the left of the mesosigmoid appears to be safe in the short term, as no complications or recurrence of POP were observed in the present two cases after more than 6 months postoperatively. Further follow‐up is needed to determine the long‐term safety. Based on the results of these two cases, mesh fixation to the sacral promontory from the left side of the mesosigmoid may be a safe and feasible option for patients in whom exposure from the right side is challenging due to PDM.

A limitation in the present two cases was the inability to diagnose PDM preoperatively. Hanaoka et al. retrospectively analyzed CT images of 837 patients with left‐sided colon cancer and no history of abdominal surgery, defining PDM as cases where the right edge of the descending colon was located medially to the right edge of the left kidney [7]. They found a PDM prevalence of 2.3% (19/837), comparable to the previously reported intraoperative diagnosis rate of 1.2%–2.4% [3, 4]. A review of preoperative CT images in the present two cases confirmed that the right edge of the descending colon was indeed located medially to the right edge of the left kidney (Figure 4). The definition of PDM by Hanaoka et al. serves as a useful screening method due to its simplicity and independence from image quality, slice thickness, or image reading skills. With the increasing number of sacrocolpopexy procedures for POP, preoperative diagnosis of PDM is becoming increasingly important. Preoperative evaluations should consider not only the vascular structures anterior to the sacrum but also potential intestinal positional abnormalities to optimize surgical planning and reduce intraoperative challenges.

## Consent

The authors have nothing to report.

## Conflicts of Interest

The authors declare no conflicts of interest.
